# Supplementary material for: Compact A15 Frank-Kasper nano-phases at the origin of dislocation loops in face-centred cubic metals
Source: Nat Commun. 2023 May 25;14:3003. doi: 10.1038/s41467-023-38729-6 (PMC10213062; doi:10.1038/s41467-023-38729-6)
Supplement: Supplementary file 1 — Supplementary Information [file 41467_2023_38729_MOESM1_ESM.pdf]

SUPPLEMENTARY MATERIALS

Compact A15 Frank-Kasper nano-phases at the origin of dislocation loops in face-centred cubic metals

Alexandra M. Goryaeva et al.

CONTENTS

SUPPLEMENTARY NOTE 1: Performance of EAM potentials 3
SUPPLEMENTARY NOTE 2: Elastic corrections in Ni from ML force field 4
SUPPLEMENTARY NOTE 3: Large-scale calculations in Al and Cu 9
SUPPLEMENTARY NOTE 4: Energy landscape of SIA clusters in Cu 11
SUPPLEMENTARY NOTE 5: Dislocation loop energy based on elastic theory 12
SUPPLEMENTARY NOTE 6: Relaxation volume and dipole tensors 13
SUPPLEMENTARY NOTE 7: Observation of interstitial dislocation loops in TEM 14

Supplementary References 15

SUPPLEMENTARY NOTE 1: Performance of EAM potentials

Large-scale atomistic calculations of Frenkel pair accumulation (FPA) and cascades in Al and Cu were performed using semi-empirical EAM potentials by Mendelev et al. [24] and by Mishin et al. [25], respectively. These potentials are numerically fast and provide a good qualitative agreement with DFT calculations. For fcc Ni, there is no suitable semi-empirical potential that correctly reproduces the relative stability of small SIA clusters. Thus, no large-scale simulations were performed for Ni.

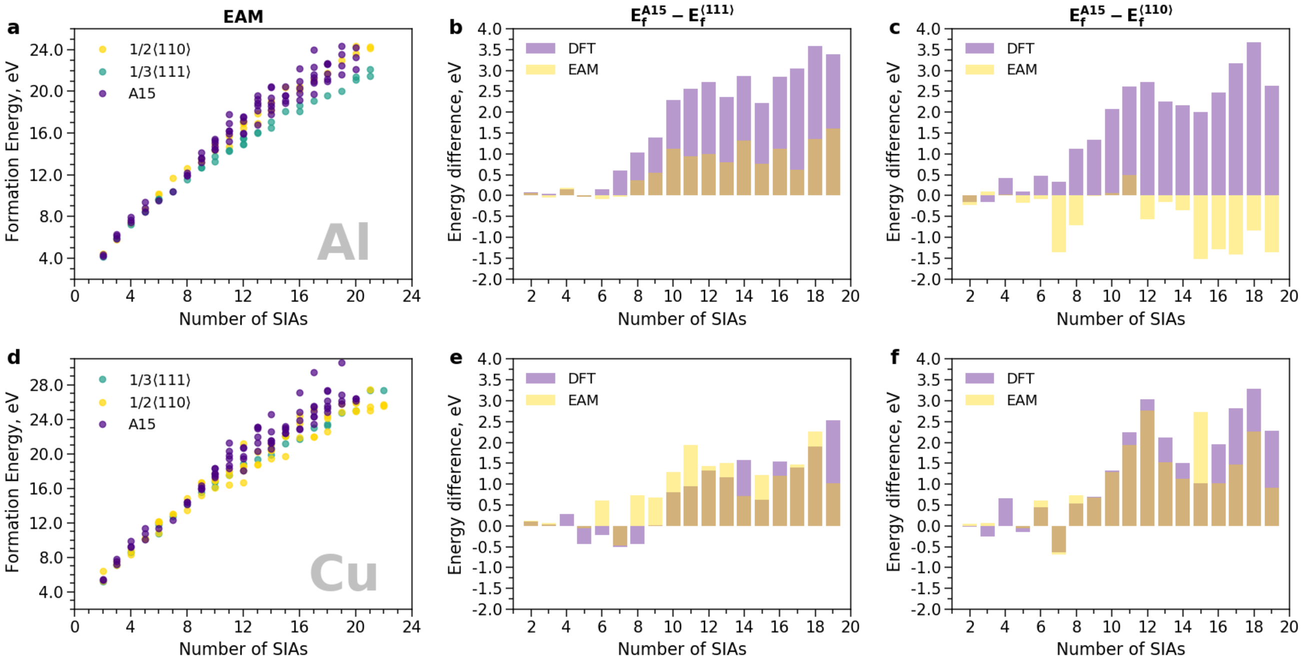

Supplementary Figure 1: Formation energies and relative stability of A15, $\frac{1}{3}\langle 111\rangle$ and $\frac{1}{2}\langle 110\rangle$ interstitial clusters. The upper and lower panels provide the energies in fcc Al and fcc Cu, respectively. Subplots (a,d) provide the formation energies from EAM potentials. Subplots (b,c,e,f) illustrate the relative stability of A15 clusters with respect to $\frac{1}{3}\langle 111\rangle$ (b,e) and $\frac{1}{2}\langle 110\rangle$ loops (c,f) obtained with DFT and EAM potentials. The negative energy difference indicates that A15 clusters are more stable than the 2D clusters.

Supplementary Figure 1 provides the formation energies for the three SIA cluster types as provided by the EAM potentials and compares these energies with DFT results (Figure 2 in the main text). Compared to DFT, EAM potential for Al provides higher formation energies of $\frac{1}{2}\langle 110\rangle$ loop family and lower formation energies of large A15. In fcc Cu, the relative stability provided by DFT and EAM is in good agreement. Overall, the calculations performed using DFT and semi-empirical force fields provide qualitatively similar energy landscapes of small A15 clusters and most stable dislocation loops, which allows using the EAM potentials for large-scale molecular dynamics simulations.

It is worth noting that in Al, Frank dislocation loops $\frac{1}{3}\langle 111\rangle$ is the most stable 2D defect family while in Cu, prismatic $\frac{1}{2}\langle 110\rangle$ loops are more stable than Frank loops (till *ca* $N = 30$, see Supplementary Figure.2).

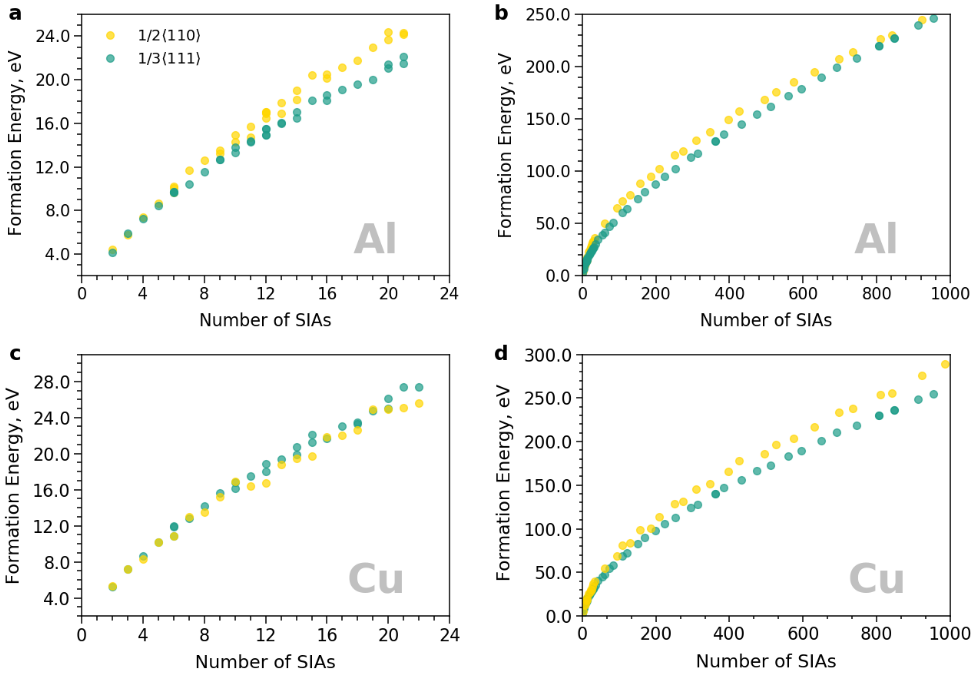

Supplementary Figure 2: Formation energies of small (a, c) and large (b, d) dislocation loops in fcc Al and Cu computed with EAM potentials [24, 25]

SUPPLEMENTARY NOTE 2: Elastic corrections in Ni from ML force field

Compared to Al and Cu, Ni has bigger elastic constants and higher formation energy of SIAs. For all three types of defects considered in this work, the formation energy is almost twice as large (see Fig. 2 of the main text) in Ni than in Al and Cu. In addition to that, due to the finite size of simulation cells in *ab initio* calculations (1,372 atoms in this work), the elastic corrections to formation energies (computed using elastic dipole tensor) [32] reach up to 4 eV for 20 SIAs in Ni. Such high values call into question the pertinence of the elastic corrections computed for the defect clusters with more than 10 SIAs. To address this point, one can use a semi-empirical potential (EAM, MEAM) that is in agreement with the DFT formation energies of A15, $\frac{1}{3}\langle 111\rangle$, and $\frac{1}{2}\langle 110\rangle$ defects up to 20 SIA. Then, in much larger boxes than those used in DFT, we can estimate the elastic corrections by making the difference between the formation energy of defects in large boxes and the boxes with the same size as the DFT calculations. After testing many existing potentials, we did not find a suitable one. Therefore, we develop an accurate machine learning (ML) force field that will allow us to use the strategy described above.

Fitting accurate ML potentials

Here we describe a development procedure of interatomic ML potentials suitable for the study of SIA defects, and their clusters in fcc Ni. We construct the potentials using linear (LML) [11, 13], quadratic (QNML) [11], and kernel (KNML) input-output mapping from descriptor space. In contrast to classical force fields, where the performance and limitations of the potential are mainly determined by the physical formalism, the performance and accuracy of ML potentials are dependent on three

Supplementary Table 1: DFT database used for the development of ML potentials of fcc Ni. The `DB class` column gives a short description of the calculations, the `Atoms per cell` column lists the number of atoms in the cell used for DFT calculations, the `Properties to fit` column corresponds to the fitted properties for training/testing (energy E, force F, stress S), the fourth column corresponds to the total number of train/test data that are used to fit/test the potential n_E, n_F, n_S stands for a number of energies, forces, and stresses, respectively, included in the fit. Finally, the last column `Configurations train/test` gives the total number of configurations that were used for the train/test purposes.

Ni Database					
DB class	Content	Atoms per cell	Properties to fit	$n_E + n_F + n_S$ train/test	Configurations train/test
1	Elasticity (fcc)	4	ES	273/0	39/0
2	A15 clusters, 0 K	1374-1396	EFS	248,640/ 74,592	60/18
3	A15 clusters, random noise	1379	EFS	24,864/ 0	6/0
4	$\frac{1}{2}\langle 110\rangle$ loops, 0 K	1374-1396	EFS	103,600/41,440	25/10
5	$\frac{1}{2}\langle 110\rangle$ loops, random noise	1374-1396	EFS	24,864/ 0	6/0
6	$\frac{1}{3}\langle 111\rangle$ loops, 0 K	1374-1396	EFS	136,752/33,152	33/8
7	$\frac{1}{3}\langle 111\rangle$ loops, random noise	1379	EFS	24,864/ 0	6/0
8	MD - liquid (2,000 K $\rightarrow$ 3,000 K)	256	EFS	11,625/3,875	15/5
9	MD - bcc bulk (100 K $\rightarrow$ 2,000 K)	256	EFS	13,175/3,110	17/4
10	MD - A15 (100 K $\rightarrow$ 2,000 K)	1379	EFS	33,152/12,432	8/3
Total				621,809 / 168,601	215 / 48

equally important components: the database, its representation in descriptor space, and the regression algorithm.

Aiming to develop ML potentials for calculations of elastic corrections for SIA defects, we include in the database the 0 K DFT configurations from Figure 2 of the main paper, as well as fcc structures deformed within the elastic regime. Further, the database is complemented by the configurations of the three defect types (from Fig. 2) where some random noise is applied on the 0 K atomic positions, MD simulations at 100 - 2,000 K, and liquid at 2,000-3,000 K. The full content of the training database is reported in Supplementary Table 1.

The procedure of fitting LML and QNML potentials is described in the previous works [11, 13]. The new kernel potential is based on the so-called kernel noise machine learning (KNML) formalism implemented in MiLaDy [14]. This model has some similarities with QNML framework [11]. Instead of performing a single regression with a fully non-linear formalism in descriptor space, first, a linear model (LML) is fitted in and then the kernel model (KML) is applied to the difference between the target value and the LML estimation. Finally, the local atomic energy can be writtn as: $\epsilon_{s,a}^{\text{KNML}} = (\mathbf{w}^{\text{LML}} \oplus \mathbf{w}^{\text{KML}})^\top [\mathbf{D}_{s,a} \oplus \mathbf{k}(\mathbf{D}_{s,a})]$, where $\mathbf{D}_{s,a}$ is a D-dimensional descriptor that encode the local atomic neighbourhood information of the ath atom from the system s within a cutoff distance R_c. Besides, $\mathbf{k}(\cdot) \in \mathbb{R}^{K\times 1}$ is a column vector that measures the distance in the descriptor space between the descriptor $\mathbf{D}_{s,a} \in \mathbb{R}^D$ and the K sparse points $\mathbf{z}_k \in \mathbb{R}^D$ $(k = 1, \ldots, K)$ selected from the database. This distance is measured using a kernel function $k(\cdot,\cdot) : \mathbb{R}^D \times \mathbb{R}^D \rightarrow \mathbb{R}$. Within this formulation, the

Supplementary Table 2: Elastic properties of fcc Ni provided by the developed LML, QNML and KNML potentials and their comparison with the reference DFT values. The fcc lattice parameter a_0 and the elastic constants are reported in Å and GPa, respectively. MAEs denote the corresponding fitting mean squared error for the total energy of each box (MAE_E, in meV), energy per atom (MAE_ϵ in meV), forces (MAE_F, in meV/Å) and stress (MAE_S, in meV/Å^3).

	Ni			
	LML	QNML	KNML	DFT
a_0	3.5236	3.5235	3.5234	3.5234
B	193.3	192.8	193.5	193.9
C_{11}	274.4	273.2	271.5	270.3
C_{12}	152.7	152.6	154.5	155.8
C_{44}	126.3	126.9	127.8	127.2
MAE_E	293	210	171	
MAE_ϵ	1	0.8	0.1	
MAE_F	30	28	7	
MAE_S	35	23	17	

parameters of LML and KML parts are in direct product $\mathbf{w} = \mathbf{w}^{\text{LML}} \oplus \mathbf{w}^{\text{KML}}$, meaning that there are $1 + D + K$ parameters in total. This can be interpreted as an extension of K components to the original D-dimensional descriptor space. The energy descriptor becomes $\sum_{a \in s}[\mathbf{D}_{s,a} \oplus \mathbf{k}(\mathbf{D}_{s,a})]$, from which the forces and stresses can be derived [3–5, 13].

The multidimensional representation of the local atomic environments is performed using bispectrum SO(4) [4] with a cutoff distance $R_c = 5.0$ Å and angular moment $j_{\text{max}} = 5$, resulting in a descriptor representation of dimension $D = 91$. For the KNML approach, we have selected $K = 5,277$ sparse-points using the standard CUR [22] approach, as implemented in MiLaDy [14].

All three potentials are in good agreement with the DFT database (Supp. Tab. 2). The errors of fit are lower than 1 meV/atom, and even reach 0.1 meV/atom for KNML potential. Furthermore, the targeted properties such as lattice parameter and elastic constants are very well reproduced (summarized in Supp. Tab. 2). Further, using the two potentials with the lowest errors, QNML and KNML, we compute the formation energies and relative stability of SIA defects in fcc Ni and compare them with DFT results (Supp. Fig. 3) It is worth noting that ML and DFT simulations in Supp. Fig. 3 were performed using the same conditions, *i.e.* 0 K structural relaxation in a box of size $7a_0 \times 7a_0 \times 7a_0$, where a_0 is the cubic lattice parameter of the fcc lattice (the values are presented in Supp. Tab. 2). Both ML results are very close to the DFT counterparts, however, for further calculations of the elastic corrections we will use only the most accurate potential, namely KNML.

Calculations of elastic corrections

The elastic corrections are estimated as the difference between the formation energy of defects in a large $20a_0 \times 20a_0 \times 20a_0$ box with 32,000 atoms, and a $7a_0 \times 7a_0 \times 7a_0$ box with 1,372 $\pm N$

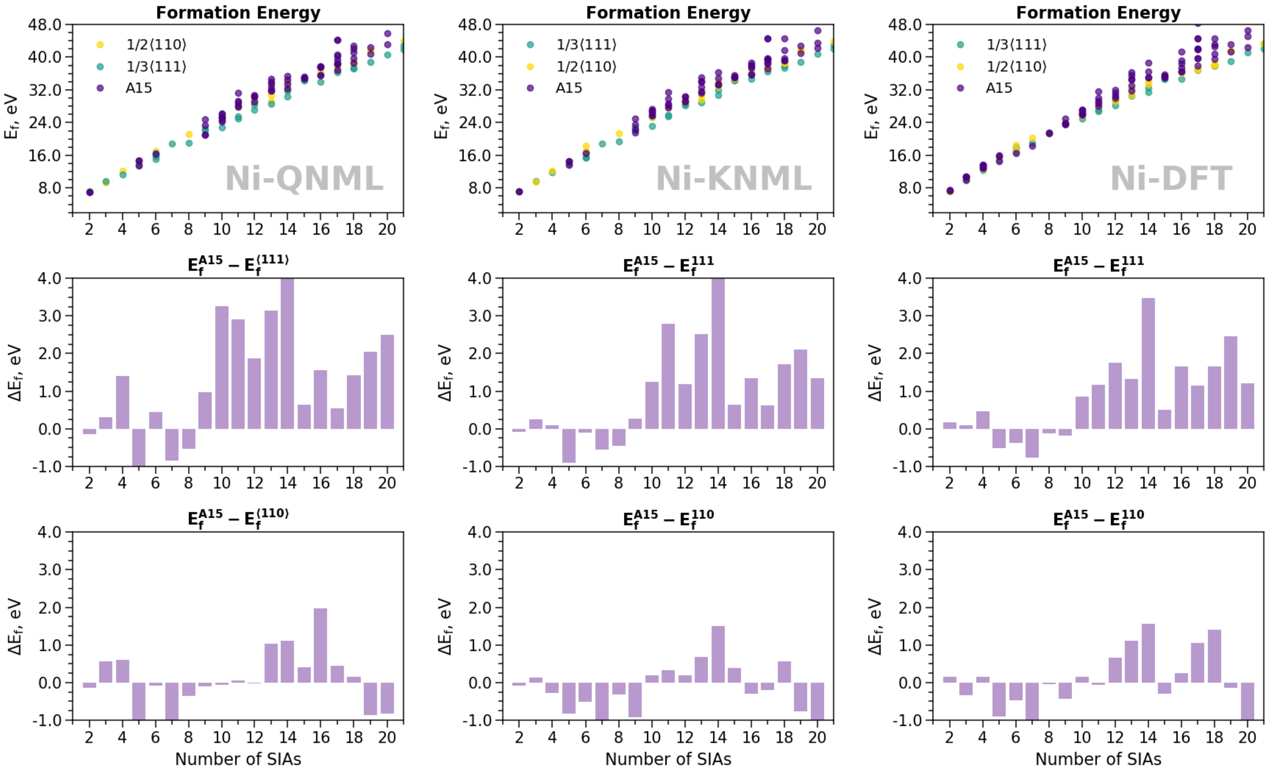

Supplementary Figure 3: Relative stability of $\frac{1}{2}\langle 110\rangle$, $\frac{1}{3}\langle 111\rangle$ and A15 small clusters computed in fcc Ni using QNML (first column) and KNML (second column) potentials, and DFT (third column). The energies are provided without elastic corrections from the simulations in $7a_0 \times 7a_0 \times 7a_0$ boxes with 1,372 $\pm$ N atoms, where a_0 is the corresponding DFT/ML lattice constant; N is the number of interstitial atoms. The type of simulations is indicated on each subplot.

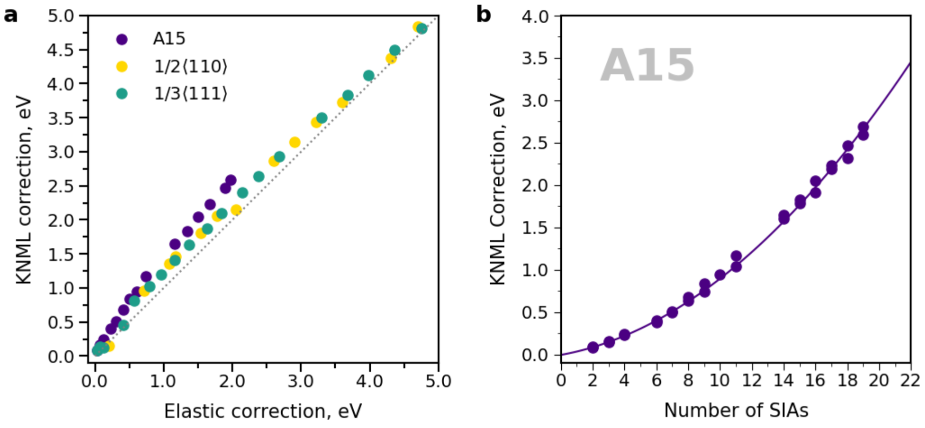

Supplementary Figure 4: Elastic corrections computed for the different types of SIA clusters in fcc Ni for the $7a_0 \times 7a_0 \times 7a_0$ simulation cell. (a) Comparison of the corrections provided by the KNML potential and classic elastic formulation based on dipole tensors [32]. (b) Elastic corrections for A15 clusters as a function of defect size.

atoms, similar to those in DFT calculations. Supplementary Figure 4 reports the elastic corrections for the 2D dislocation loops and 3D A15 clusters. For the 2D loops, even at large sizes, there is a

remarkable agreement between elastic theory and the present procedure based on ML potentials. For A15 clusters, the agreement is good only for small cluster sizes. The difference between the ML model and elastic theory [32] increases with the size of the clusters. This difference is likely related to the internal pressure or relaxation around central atoms of the nano-phase (as described in the Discussion section of the main text). These effects cannot be taken into account by the classic formulation of elastic corrections [32].

Consequently, for accurate corrections of our DFT calculations of A15 clusters in Ni (Fig.2 of the main text), we use the present ML corrections.

SUPPLEMENTARY NOTE 3: Large-scale calculations in Al and Cu

Frenkel pair accumulation

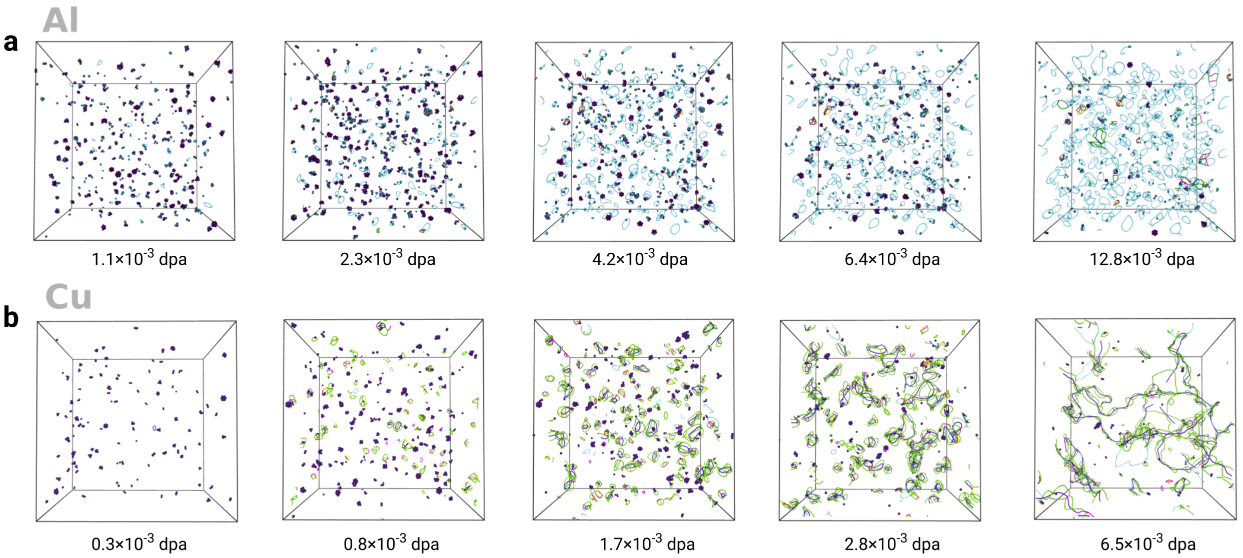

Supplementary Figure 5: Evolution of the microstructue in (a) fcc Al and (b) fcc Cu during the Frenkel Pair accumulation simulations. The dose is indicated below each snapshot. A15 clusters are shown with purple atoms, $\frac{1}{3}\langle 111\rangle$ dislocations are cyan, $\frac{1}{2}\langle 110\rangle$ dislocations are blue, $\frac{1}{6}\langle 211\rangle$ Shockley dislocations are green, $\frac{1}{3}\langle 001\rangle$ dislocations are yellow, other dislocation types are indicated with red lines. A15 clusters are detected using distortion score [12], and dislocations are identified using DXA analysis in OVITO [28]. The perfect prismatic loops (shown with dark blue) are commonly identified by DXA as partial Shockley dislocations that form perfect prismatic loops according to the reaction $\frac{1}{6}\langle 211\rangle + \frac{1}{6}\langle 12\bar{1}\rangle \rightarrow \frac{1}{2}\langle 110\rangle$.

Displacement cascades

The molecular Dynamics (MD) calculations of displacement cascades take into account long-range diffusion processes and complement the FPA simulations in Al and Cu.

In fcc Al, the calculations of displacement cascades are performed with the primary knock-on atom (PKA) energy of 50 keV at 100 K in a simulation box with 3,764,768 atoms. The simulations are carried out using DYMOKA [6] in the microcanonical ensemble with periodic boundary conditions. The time step was adjusted during the simulations and no electronic losses were considered. Before initiating the displacement cascade, the system of particles was equilibrated for 3 ps at the chosen temperature. The PKA direction is $\langle 135\rangle$, which is a reasonable representation of average behavior. The equilibrium EAM potential [20] has been used after being hardened. Pair interactions at short distances are described with ZBL potential, with a transition to the equilibrium potential between 1.7 Åand 2.0 Å.

In fcc Cu, we analyze the structural damage produced by 100 keV displacement cascade at 300 K in a simulation box with 5,324,000 atoms. These Cu structures are taken from the dataset R093 [26] of the open-source database cascadesdb.org by the International Atomic Energy Agency (IAEA).

Both intrinsic and extrinsic dislocation loops are formed in the centre of cascades. In addition to

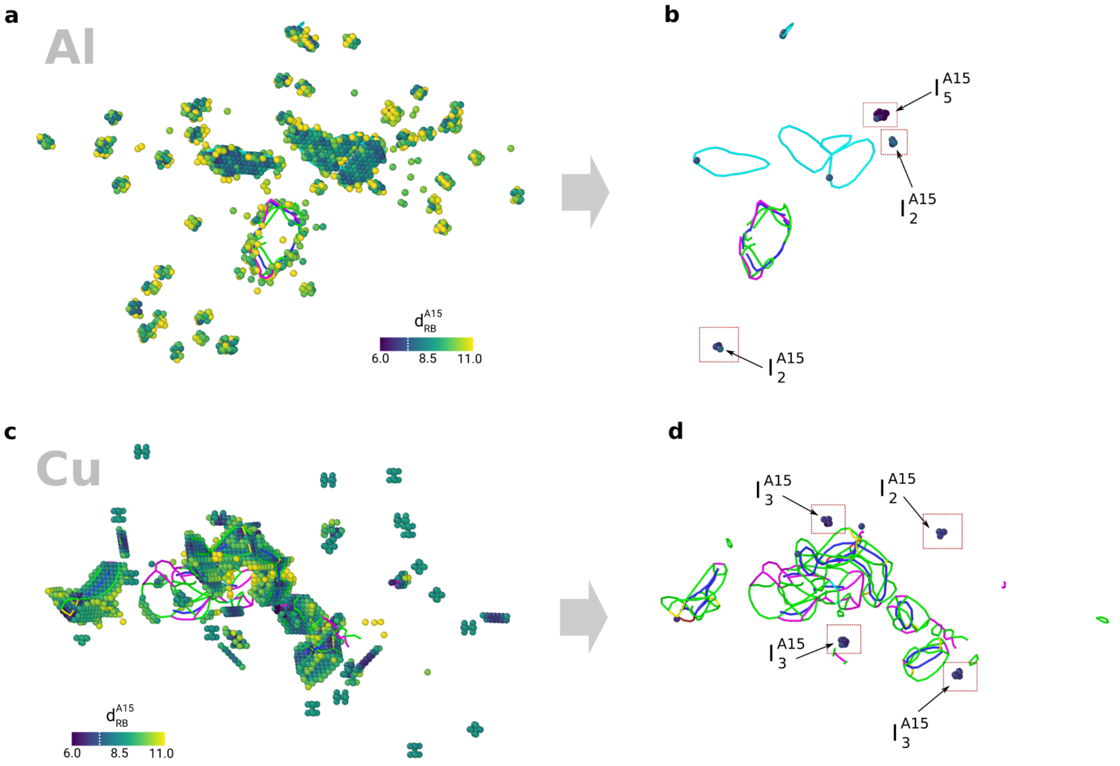

Supplementary Figure 6: Structural damage produced by displacement cascades in fcc Al and Cu. (a,b) 50 keV cascade at 100 K in fcc Al. (c,d) 100 keV cascade in fcc Cu at 300 K. (a, c) All the interstitial-type defects and dislocations produced by the cascade; (b, d) A15 interstitial clusters and dislocation lines in this area. The $\frac{1}{3}\langle 111\rangle$ Frank dislocations are shown in light blue, $\frac{1}{6}\langle 112\rangle$ Shockley dislocations are green, $\frac{1}{2}\langle 110\rangle$ dislocations are dark blue, other dislocation types are shown in red. The atoms are coloured according to the distance d_{RB}^{A15} that describes the proximity of atomic environments to the A15 SIA clusters.

the commonly observed Frank and prismatic loops [1, 8, 26], few A15 interstitial clusters are detected in the cloud of structural damage at the periphery of the cascades using the distortion scores of local atomic environments (see the Methods in the main text). Those clusters are indicated with red rectangles in Supp. Fig. 6b,d. In fcc Cu, previous studies of displacement cascades [2, 27, 33] report the presence of sessile SIA clusters in irregular 3D configurations. The I_3^{A15} and I_4^{A15} clusters are similar to small irregular 3D clusters previously observed in low temperature 5 - 10 keV cascades in Refs. [2, 27, 33]. The large 3D SIA clusters (up to 46 atoms) formed in 25 keV cascades [33] were shown to transform in prismatic $\frac{1}{2}\langle 110\rangle$ loops after MD annealing. This behaviour is similar to that of A15 clusters observed in our study. The number of A15 clusters detected in this work per cascade is small and represents only 4-5 % of interstitial atoms. This observation is consistent with the number of 3D clusters with C15 structure in bcc Fe produced in displacement cascades [7, 23]. Although not numerous, the sessile 3D clusters can act as a source of dislocation loops in the further evolution of the microstructure. The investigations of C15 clusters in bcc Fe, that form only 5 % of SIAs clusters in a single cascade, were shown to have a major impact in the overlapping cascades [7]. In perspective, it will be interesting to explore the effect of A15 clusters in fcc metals on the population of dislocation loops in overlapping cascades.

SUPPLEMENTARY NOTE 4: Energy landscape of SIA clusters in Cu

This section provides the full disconnectivity graph of I_8 energy landscape in Cu computed with TAMMBER [29–31]. The representative subset is shown in Figure 6c of the main text.

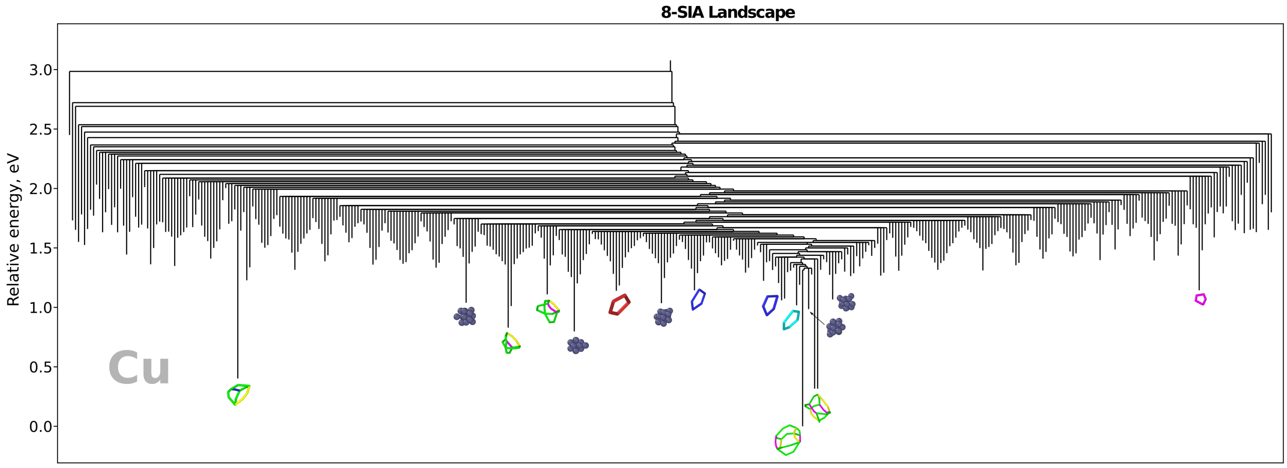

Supplementary Figure 7: Disconnectivity graph between different states of I_8 clusters in fcc Cu. A15 clusters are detected using distortion scores [12], and dislocations lines are identified using DXA analysis [28]. A15 clusters are shown with purple atoms, $\frac{1}{3}\langle 111\rangle$ faulted Frank loop is cyan, $\frac{1}{2}\langle 110\rangle$ loops are blue, $\frac{1}{6}\langle 411\rangle$ loop is red, $\frac{1}{6}\langle 112\rangle$ Shockley dislocations are green. The lowest energy dislocation configurations are built by partial Shockley dislocations that form perfect prismatic loops according to the reaction $\frac{1}{6}\langle 211\rangle + \frac{1}{6}\langle 12\bar{1}\rangle \rightarrow \frac{1}{2}\langle 110\rangle$.

SUPPLEMENTARY NOTE 5: Dislocation loop energy based on elastic theory

According to the elastic theory of dislocations [16], the formation energy of a dislocation loop is essentially related to the two quantities: (i) the elastic energy of the dislocation that encloses the interstitial cluster and (ii) the stacking fault energy. The elastic contribution (i) E_{el} to the formation energy contains the energy stored in the elastic field of the loop and the core energy of the dislocation that encloses the cluster:

$$E_{el} = 2\pi R^* K_a \ln\left(\frac{R^*}{\delta}\right) + 2\pi R^* E_{\delta-c} + \gamma\pi R^{*2}, \tag{1}$$

where R^* (determined by the number N of SIAs) is the radius of an equivalent circular loop with the same perimeter; $E_{\delta-c}$ is the core energy of the surrounding dislocation enclosed in a cylinder centred on dislocation of radius δ (usually taken as 2 times the Burgers vector $\mathbf{b}$ of the dislocation); γ is the average stacking fault energy of the loop; K_a is the anisotropic elastic modulus, which depends on the Burgers vector $\mathbf{b}$ (scales as b^2) and the habit plane can be evaluated solely from the elastic tensor of the fcc matrix [16]. The shortest Burgers vector with translational symmetry in fcc matrix is $\frac{1}{2}\langle 110\rangle$. Prismatic $\frac{1}{2}\langle 110\rangle$ dislocation loops ($\gamma = 0$) are commonly observed in a large-size interstitial defect in fcc metals [10, 17–19]. However, at intermediate sizes, it is more favorable for the system (Eq. 1) to arrange into faulted loops ($\gamma > 0$) surrounded by immobile dislocations having shorter Burgers vector $\frac{1}{3}\langle 111\rangle$ [10, 17–19].

Our DFT and EAM calculations, up to N = 20-30 (Supp. Fig. 2, Fig. 2 in the main text), indicate that small-size loop morphologies in Al and Cu are different. The preferred types of small loops in Al are immobile faulted Frank $\frac{1}{3}\langle 111\rangle$ loops, while crowdions $\frac{1}{2}\langle 110\rangle$ are more stable in Cu.

SUPPLEMENTARY NOTE 6: Relaxation volume and dipole tensors

Supplementary Table 3: Relaxation volumes Ω^R (in Ω_0 units) for 1-SIA and 7-SIA clusters, relaxation volume tensors Ω_{ij} (in Ω_0 units) and elastic dipole tensors P_{ij} (in eV) for 7-SIA clusters. The values for 7-SIAs are from DFT performed in this work, the 1-SIA values are taken from Ref. [21]

		1-SIA Ω^R	7-SIA Ω^R	7-SIA Ω_{ij}	7-SIA P_{ij} (eV)
Cu	A15		10.80	$\begin{bmatrix} 3.60 & 0.00 & 0.00 \\ 0.00 & 3.60 & 0.00 \\ 0.00 & 0.00 & 3.60 \end{bmatrix}$	$\begin{bmatrix} 111.68 & 0.00 & 0.00 \\ 0.00 & 111.68 & 0.00 \\ 0.00 & 0.00 & 111.68 \end{bmatrix}$
	$\frac{1}{3}\langle 111\rangle$	1.85 [21]	11.06	$\begin{bmatrix} 3.48 & 0.77 & 0.77 \\ 0.77 & 3.79 & 0.85 \\ 0.77 & 0.85 & 3.79 \end{bmatrix}$	$\begin{bmatrix} 108.01 & 23.90 & 23.90 \\ 23.90 & 117.47 & 26.39 \\ 23.90 & 26.39 & 117.47 \end{bmatrix}$
	$\frac{1}{2}\langle 110\rangle$	1.82 [21]	11.07	$\begin{bmatrix} 3.66 & 2.21 & 0.01 \\ 2.21 & 3.66 & 0.01 \\ 0.01 & 0.01 & 3.75 \end{bmatrix}$	$\begin{bmatrix} 113.56 & 68.43 & 0.24 \\ 68.43 & 113.56 & 0.24 \\ 0.24 & 0.24 & 116.21 \end{bmatrix}$
Al	A15		11.76	$\begin{bmatrix} 3.92 & 0.00 & 0.00 \\ 0.00 & 3.92 & 0.00 \\ 0.00 & 0.00 & 3.92 \end{bmatrix}$	$\begin{bmatrix} 93.48 & 0.00 & 0.00 \\ 0.00 & 93.48 & 0.00 \\ 0.00 & 0.00 & 93.48 \end{bmatrix}$
	$\frac{1}{3}\langle 111\rangle$	2.51 [21]	12.42	$\begin{bmatrix} 4.14 & 0.93 & 0.93 \\ 0.93 & 4.14 & 0.93 \\ 0.93 & 0.93 & 4.14 \end{bmatrix}$	$\begin{bmatrix} 98.80 & 22.18 & 22.18 \\ 22.18 & 98.90 & 22.23 \\ 22.18 & 22.23 & 98.90 \end{bmatrix}$
	$\frac{1}{2}\langle 110\rangle$	2.46 [21]	12.39	$\begin{bmatrix} 4.32 & 1.72 & 0.07 \\ 1.72 & 4.32 & 0.07 \\ 0.07 & 0.07 & 3.75 \end{bmatrix}$	$\begin{bmatrix} 103.21 & 41.05 & 1.58 \\ 41.05 & 103.21 & 1.58 \\ 1.58 & 1.58 & 89.54 \end{bmatrix}$
Ni	A15		11.10	$\begin{bmatrix} 3.70 & 0.00 & 0.00 \\ 0.00 & 3.70 & 0.00 \\ 0.00 & 0.00 & 3.70 \end{bmatrix}$	$\begin{bmatrix} 146.74 & 0.00 & 0.00 \\ 0.00 & 146.74 & 0.00 \\ 0.00 & 0.00 & 146.74 \end{bmatrix}$
	$\frac{1}{3}\langle 111\rangle$	1.86 [21]	11.18	$\begin{bmatrix} 3.44 & 0.82 & 0.82 \\ 0.82 & 3.87 & 1.08 \\ 0.82 & 1.08 & 3.87 \end{bmatrix}$	$\begin{bmatrix} 136.56 & 32.34 & 32.34 \\ 32.34 & 153.46 & 42.99 \\ 32.34 & 42.99 & 153.46 \end{bmatrix}$
	$\frac{1}{2}\langle 110\rangle$	1.87 [21]	11.35	$\begin{bmatrix} 3.77 & 2.61 & 0.08 \\ 2.61 & 3.77 & 0.08 \\ 0.08 & 0.08 & 3.81 \end{bmatrix}$	$\begin{bmatrix} 149.41 & 103.63 & 3.14 \\ 103.63 & 149.41 & 3.14 \\ 3.14 & 3.14 & 151.08 \end{bmatrix}$

SUPPLEMENTARY NOTE 7: Observation of interstitial dislocation loops in TEM

An electron-irradiated Ni-0.4Ti thin foil was post-characterized after irradiation using a FEI TECNAI G2 Transmission Electron Microscope (TEM) operated at 200 kV. Two dislocation loops in an area of interest were identified. TEM micrographs of the loops are taken under two-beam kinematic bright-field (KBF) condition using $g = [200]$ (Supp. Fig. 8a,b) and $g = [\bar{2}00]$ (Supp. Fig. 8c). Using the same diffraction vector, both loops "opened" after tilting from Supp. Fig. 8a to Supp. Fig. 8b. Based on the sample tilt direction shown in Supp. Fig. 8e, the inclination of loop habit plane is deduced as shown by the red and blue parallelograms respectively [9]. Then the inside-outside pair Supp. Fig. 8b,c suggests that the upper loop has inside contrast, while the lower loop has outside contrast with $g = [200]$. Therefore, based on the inside-outside method [15], we deduce that both loops are interstitial-type.

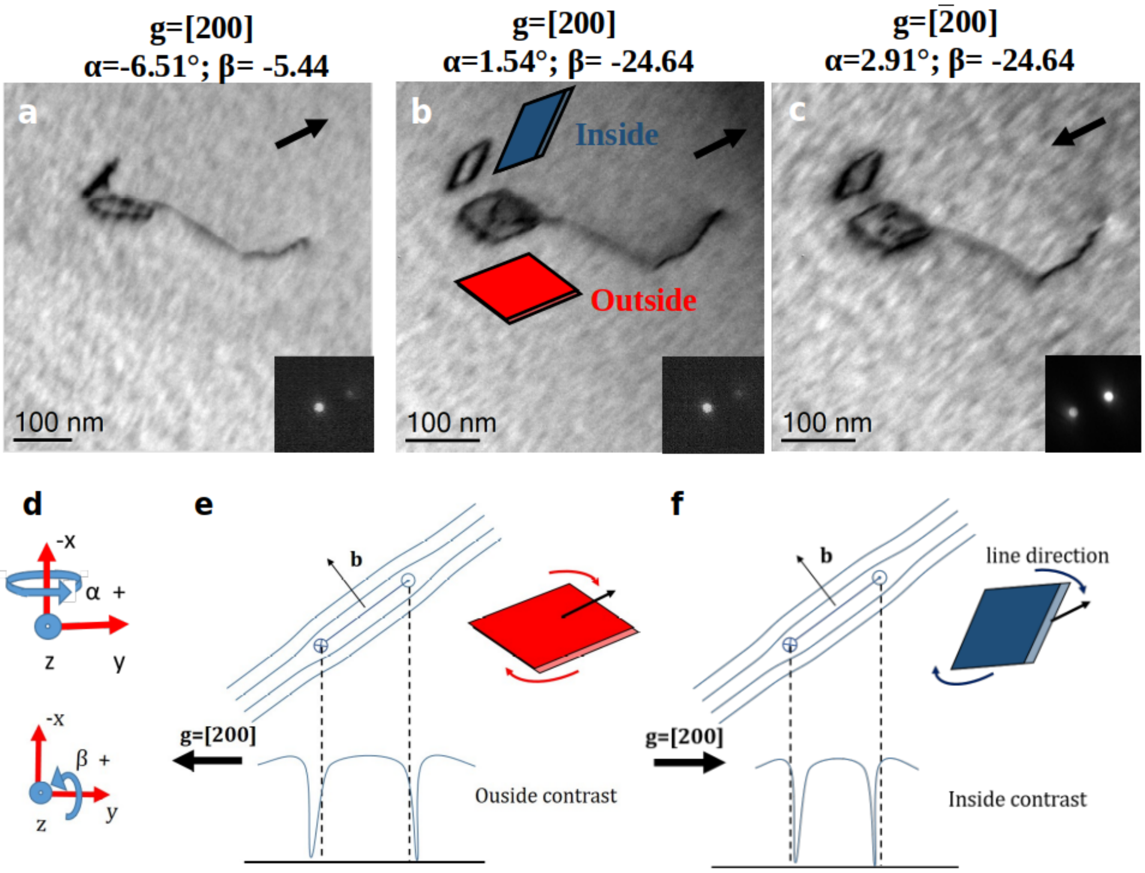

Supplementary Figure 8: Stereomicroscopy [9] applied to determine the interstitial nature of dislocation loops in electron irradiated Ni-0.4Ti. (a-c) TEM micrographs taken in two-beam Kinetics Bright Field conditions for (a, b) $g = [200]$ and (c) for $g = [\bar{2}00]$ with diffraction patterns in the corner and the direction of the diffraction vector indicated by a back arrow. The tilting direction of the α tilt and the β tilt (when α and β increases) are indicated in (d). The determination of loop nature for two loops schematized in blue and red in figure (b) is illustrated in (e), (f). The loop inclination and the direction of the diffraction vector are deduced from images (a) and (b), and the inside-outside contrast from (b) and (c) with s_g positive [15].

SUPPLEMENTARY REFERENCES

[1] D. J. Bacon, F. Gao, and Y. N. Osetsky. The primary damage state in fcc, bcc and hcp metals as seen in molecular dynamics simulations. *J. Nucl. Mater.*, 276(1-3):1, 2000.

[2] D. J. Bacon, Y. N. Osetsky, R. Stoller, and R. Voskoboinikov. MD description of damage production in displacement cascades in Copper and α-Iron. *J. Nucl. Mater.*, 323(2):152 – 162, 2003. Proceedings of the Second IEA Fusion Materials Agreement Workshop on Modeling and Experimental Validation.

[3] A. P. Bartók, S. De, C. Poelking, N. Bernstein, J. R. Kermode, G. Csányi, and M. Ceriotti. Machine learning unifies the modeling of materials and molecules. *Sci. Adv.*, 3(12):e1701816, 2017.

[4] A. P. Bartók, R. Kondor, and G. Csányi. On representing chemical environments. *Phys. Rev. B*, 87:184115, 2013.

[5] A. P. Bartók, M. C. Payne, R. Kondor, and G. Csányi. Gaussian approximation potentials: The accuracy of quantum mechanics, without the electrons. *Phys. Rev. Lett.*, 104:136403, 2010.

[6] C. S. Becquart, K. M. Decker, C. Domain, J. Ruste, Y. Souffez, J. C. Turbatte, and J. C. V. Duysen. Massively parallel molecular dynamics simulations with eam potentials. *Radiat. Eff. Defects Solids.*, 142(1-4):9–21, 1997.

[7] J. Byggmästar, F. Granberg, A. E. Sand, A. Pirttikoski, R. Alexander, M.-C. Marinica, and K. Nordlund. Collision cascades overlapping with self-interstitial defect clusters in Fe and W. *J. Phys.: Condens. Matter*, 31:245402, 2019.

[8] A. F. Calder, D. Bacon, A. Barashev, and Y. N. Osetsky. On the origin of large interstitial clusters in displacement cascades. *Philos. Mag.*, 90(7-8):863–884, 2010.

[9] J. W. Edington and J. Edington. *Interpretation of transmission electron micrographs.* Springer, 1975.

[10] J. W. Edington and R. E. Smallman. Faulted dislocation loops in quenched aluminium. *Philos. Mag. A*, 11(114):1109–1123, 1965.

[11] A. M. Goryaeva, J. Dérés, C. Lapointe, P. Grigorev, T. D. Swinburne, J. R. Kermode, L. Ventelon, J. Baima, and M.-C. Marinica. Efficient and transferable machine learning potentials for the simulation of crystal defects in bcc Fe and W. *Physical Review Materials*, 5(10):103803, Oct. 2021.

[12] A. M. Goryaeva, C. Lapointe, C. Dai, J. Dérès, J.-B. Maillet, and M.-C. Marinica. Reinforcing materials modelling by encoding the structures of defects in crystalline solids into distortion scores. *Nat. Commun.*, 11:4691, 2020.

[13] A. M. Goryaeva, J.-B. Maillet, and M.-C. Marinica. Towards better efficiency of interatomic linear machine learning potentials. *Comp. Mater. Sci.*, 166:200 – 209, 2019.

[14] A. M. Goryaeva and M. C. Marinica. *`MiLaDy - Machine Learning Dynamics` code.* CEA, Saclay, 2015 – 2023.

[15] P. B. Hirsch. *Electron microscopy of thin crystals.* Plenum Press, New York, 1967.

[16] J. P. Hirth and J. Lothe. *Theory of dislocations.* Wiley, New York, 1982.

[17] D. Hull and D. J. Bacon. *Introduction to Dislocations.* Butterworth-Heinemann, Amsterdam, 2011.

[18] M. Jenkins and M. Kirk. *Characterisation of Radiation Damage by Transmission Electron Microscopy.* Series in Microscopy in Materials Science. CRC Press, 2000.

[19] S. Jitsukawa and K. Hojou. Effect of temperature and flux change on the behavior of radiation induced dislocation loops in pure Aluminum. *J. Nucl. Mater.*, 212-215:221–225, 1994.

[20] X.-Y. Liu, F. Ercolessi, and J. B. Adams. Aluminium interatomic potential from density functional theory calculations with improved stacking fault energy. *Model. Simul. Mater. Sci. Eng.*, 12(4):665–670, 2004.

[21] P.-W. Ma and S. L. Dudarev. Nonuniversal structure of point defects in face-centered cubic metals. *Phys. Rev. Materials*, 5:013601, 2021.

[22] M. W. Mahoney and P. Drineas. CUR matrix decompositions for improved data analysis. *Proceedings of the National Academy of Sciences*, 106(3):697–702, Jan. 2009.

[23] M.-C. Marinica, F. Willaime, and J.-P. Crocombette. Irradiation-induced formation of nanocrystallites with C15 laves phase structure in bcc Iron. *Phys. Rev. Lett.*, 108:025501, 2012.

[24] M. Mendelev, M. Kramer, C. Becker, and M. Asta. Analysis of semi-empirical interatomic potentials appropriate for simulation of crystalline and liquid Al and Cu. *Philos. Mag.*, 88(12):1723–1750, 2008.

[25] Y. Mishin, M. J. Mehl, D. A. Papaconstantopoulos, A. F. Voter, and J. D. Kress. Structural stability and lattice defects in Copper: Ab initio, tight-binding, and embedded-atom calculations. *Phys. Rev. B*, 63:224106, 2001.

[26] K. Nordlund, S. Zinkle, A. Sand, F. Granberg, R. Averback, R. Stoller, T. Suzudo, T. Suzudo, L. Malerba, F. Banhart, W. Weber, F. Willaime, S. Dudarev, and D. Simeone. Improving atomic displacement and replacement calculations with physically realistic damage models. *Nat. Commun.*, 9:1084, 2018.

[27] Y. N. Osetsky and D. J. Bacon. Defect cluster formation in displacement cascades in Copper. *Nucl. Instrum. Meth. B*, 180(1):85 – 90, 2001. Computer Simulation of Radiation Effects in Solids.

[28] A. Stukowski, V. V. Bulatov, and A. Arsenlis. Automated identification and indexing of dislocations in crystal interfaces. *Model. Simul. Mater. Sci. Eng.*, 20(8):085007, 2012.

[29] T. D. Swinburne and D. Perez. Self-optimized construction of transition rate matrices from accelerated atomistic simulations with bayesian uncertainty quantification. *Phys. Rev. Mater.*, 2:053802, 2018.

[30] T. D. Swinburne and D. Perez. Automated calculation and convergence of defect transport tensors. *NPJ Comp. Mater.*, 6(1):190, 2020.

[31] T. D. Swinburne and D. Perez. `TAMMBER-ParSplice` code, 2020.

[32] C. Varvenne, F. Bruneval, M.-C. Marinica, and E. Clouet. Point defect modeling in materials: Coupling ab initio and elasticity approaches. *Phys. Rev. B*, 88(13):134102, 2013.

[33] R. E. Voskoboinikov, Y. N. Osetsky, and D. J. Bacon. Computer simulation of primary damage creation in displacement cascades in Copper. I. Defect creation and cluster statistics. *J. Nucl. Mater.*, 377(2):385 – 395, 2008.
